# Educational Attainment and Lifestyle Risk Factors Associated With All-Cause Mortality in the US

**DOI:** 10.1001/jamahealthforum.2022.0401

**Published:** 2022-04-08

**Authors:** Klajdi Puka, Charlotte Buckley, Nina Mulia, Aurélie M. Lasserre, Jürgen Rehm, Charlotte Probst

**Affiliations:** Institute for Mental Health Policy Research, Centre for Addiction and Mental Health, Toronto, Ontario, Canada (Puka, Lasserre, Rehm, Probst); Campbell Family Mental Health Research Institute, Centre for Addiction and Mental Health, Toronto, Ontario, Canada (Puka, Rehm, Probst); Department of Automatic Control and Systems Engineering, University of Sheffield, Sheffield, United Kingdom (Buckley); Alcohol Research Group, Public Health Institute, Emeryville, California (Mulia); Institute of Clinical Psychology and Psychotherapy, Technische Universität Dresden, Dresden, Germany (Rehm); Center for Interdisciplinary Addiction Research, Department of Psychiatry and Psychotherapy, University Medical Center Hamburg-Eppendorf, Hamburg, Germany (Rehm); Program on Substance Abuse and World Health Organization Collaborating Centres, Public Health Agency of Catalonia, Barcelona, Spain (Rehm); Dalla Lana School of Public Health and Department of Psychiatry, University of Toronto, Toronto, Ontario, Canada (Rehm); I. M. Sechenov First Moscow State Medical University (Sechenov University), Moscow, Russian Federation (Rehm); Department of Psychiatry, University of Toronto, Toronto, Ontario, Canada (Rehm, Probst); Heidelberg Institute of Global Health, Medical Faculty and University Hospital, Heidelberg University, Heidelberg, Germany (Probst).

## Abstract

**IMPORTANCE:**

The US has experienced increasing socioeconomic inequalities and stagnating life expectancy. Past studies have not disentangled 2 mechanisms thought to underlie socioeconomic inequalities in health, differential exposure and differential vulnerability, that have different policy implications.

**OBJECTIVE:**

To evaluate the extent to which the association between socioeconomic status (SES) and all-cause mortality can be decomposed into a direct effect of SES, indirect effects through lifestyle factors (differential exposure), and joint effects of SES with lifestyle factors (differential vulnerability).

**DESIGN, SETTING, AND PARTICIPANTS:**

This nationwide, population-based cohort study used the cross-sectional US National Health Interview Survey linked to the National Death Index. Civilian, noninstitutionalized US adults aged 25 to 84 years were included from the 1997 to 2014 National Health Interview Survey and were followed up until December 31, 2015. Data were analyzed from May 1 to October 31, 2021. A causal mediation model using an additive hazard and marginal structural approach was used.

**EXPOSURES:**

Both SES (operationalized as educational attainment) and lifestyle risk factors (smoking, alcohol use, obesity, and physical inactivity) were assessed using self-reported questionnaires.

**MAIN OUTCOMES AND MEASURES:**

Time to all-cause mortality.

**RESULTS:**

Participants included 415 764 adults (mean [SD] age, 49.4 [15.8] years; 55% women; 64% non-Hispanic White), of whom 45% had low educational attainment and 27% had high educational attainment. Participants were followed up for a mean (SD) of 8.8 (5.2) years during which 49 096 deaths (12%) were observed. Low educational attainment (compared with high) was associated with 83.6 (men; 95% CI, 81.8–85.5) and 54.8 (women; 95% CI, 53.4–56.2) additional deaths per 10 000 person-years, of which 66% (men) and 80% (women) were explained by lifestyle factors. Inequalities in mortality were primarily a result of greater exposure and clustering of unhealthy lifestyle factors among low SES groups; with some exceptions among women, little evidence of differential vulnerability was identified.

**CONCLUSIONS AND RELEVANCE:**

In this cohort study, differential exposure to lifestyle risk factors was an important mediator of socioeconomic inequalities in mortality. Public health interventions are needed, particularly among low SES groups, to address smoking, physical inactivity, alcohol use, and the socioenvironmental contexts within which these risk factors develop.

## Introduction

Life expectancy in the US has been stagnant or decreased during the past decade (even before COVID-19), mostly as a consequence of premature deaths from external causes, such as drug and alcohol poisonings and suicide.^1,2^ Socioeconomic inequalities in life expectancy are also pronounced in the US and have been increasing,^2,3^ likely as a result of various factors, such as lifestyle risk factors,^3,4^ exposure to environmental and occupational hazards,^5^ psychosocial stress,^6^ and access to health care.^7^ Notably, lifestyle risk factors are associated with structural and social determinants,^8^ such as environmental adversity and neighborhood quality^9,10^; availability and accessibility of alcohol,^11^ tobacco,^12^ healthy foods,^13^ and physical activity–related outlets^14^; and chronic stress.^15^ Unhealthy lifestyle factors are more prevalent among groups with lower socioeconomic status (SES),^16^ likely reflecting their greater exposure to deleterious social determinants of health behaviors, and have been shown to mediate as much as 50% of the association between low SES and all-cause mortality.^4,17–19^ In addition, effects of unhealthy lifestyle factors are more deleterious among groups with lower SES, such that greater mortality and harms are experienced by individuals with low SES even when the amount of smoking or alcohol consumption is similar or less than those with high SES.^20–23^ These findings highlight 2 main mechanisms thought to underlie socioeconomic inequalities in health: differential exposure, in that some causes of disease are unevenly distributed across socioeconomic groups (a mediation hypothesis); and differential effect or vulnerability, in that the same cause of disease can have a different effect conditional on the socioeconomic group (an interaction hypothesis).^24,25^ These mechanisms have been typically evaluated independently, even though they are not mutually exclusive and have different policy implications; it is therefore important to disentangle them.^24,25^

Causal mediation analyses^26,27^ have made it possible to disentangle differential exposure and vulnerability. Studies^28–31^ using causal mediation confirm that differential exposure and vulnerability to lifestyle risk factors independently contribute to socioeconomic inequalities in mortality. These studies have been conducted in Europe and have been limited by their evaluation of lifestyle factors one at a time,^28^ lack of data on the contributing role of each lifestyle factor,^29^ or focus on cause-specific mortality (namely, alcohol- or cardiovascular-related mortality).^30,31^ These limitations are addressed in the current study, using a large cohort from the US and indexing SES using educational attainment. We took a staged approach, first using traditional methods (evaluating educational attainment by lifestyle factor interactions) to evaluate differential vulnerability, given that this has been evaluated to a lesser extent relative to studies evaluating differential exposure. Second, we used a comprehensive model (Figure) to evaluate the extent to which the association between educational attainment and all-cause mortality can be decomposed into a direct effect of educational attainment (ie, independent of lifestyle factors and covariates), indirect effects through each lifestyle factor (differential exposure), and joint effects of educational attainment and lifestyle factors (differential vulnerability).

## Methods

### Data Source

Data came from the 1997 to 2014 National Health Interview Survey (NHIS) linked to the National Death Index (NDI), with follow-up to December 31, 2015.^32^ The NHIS is an annual, nationally representative, cross-sectional household survey of the civilian noninstitutionalized US population. Participants younger than 25 years or older than 85 years at the time of NHIS administration were removed on the assumption that they had not yet reached their final level of educational attainment (our primary exposure variable) and because their exact age was not available through the public use data files, respectively. More details on the participants and data sources are available in Table 1 and the eMethods in the Supplement. All participants in the NHIS provided written informed consent. The NHIS is approved by the Research Ethics Review Board of the National Center for Health Statistics and the US Office of Management and Budget. This study followed the Strengthening the Reporting of Observational Studies in Epidemiology (STROBE) reporting guideline and AGReMA (guideline for reporting studies of mediation analyses) reporting guideline.^33^

### Measures

The exposure, mediators, and confounders were self-reported at one time during the NHIS, whereas the outcome was obtained from the NDI. The outcome was time to all-cause mortality, operationalized as the time from the NHIS survey to death or last presumed alive. The exposure of interest (SES) was operationalized as educational attainment and categorized as low (high school diploma or less), medium (some college but no bachelor’s degree), or high (bachelor’s degree or more).^34,35^

The mediating role of alcohol use, smoking, body mass index (BMI), and physical activity was examined. Alcohol use was categorized based on the mean grams of pure alcohol consumed per day, according to the standards of the World Health Organization^36^: (1) never drinkers, (2) former drinkers, (3) category I (up to 20 g/d [women] or 40 g/d [men]), (4) category II (21–40 g/d [women] or 41–60 g/d [men]), and (5) category III (≥41 g/d [women] or ≥61 g/d [men]). Smoking was categorized as never smoker, former smoker, current some-day smoker, and current everyday smoker. Body mass index was calculated as weight in kilograms divided by height in meters squared and categorized as underweight (<18.5), healthy weight (18.5–24.99), overweight (25–29.99), or obesity (≥30).^37^ Lastly, the World Health Organization recommendations of 150 to 300 minutes of moderate-intensity physical activity per week^38^ were used to categorize physical activity as sedentary (0 min/wk), somewhat active (<150 min/wk), or active (≥150 min/wk).

With respect to confounders, all models were stratified by sex and adjusted for age (continuous), race and ethnicity, including (1) Hispanic, (2) non-Hispanic Black/African American, (3) non-Hispanic White, and (4) non-Hispanic other (American Indian or Alaska Native, Asian or Pacific Islander, and other [including multiple race and ethnicity]), marital status (married or living with partner vs never married, widowed, divorced, or separated), and survey year.

### Statistical Analysis

The analyses are detailed in the eMethods in the Supplement and were completed using the timereg package in R, version 4.1.1 (R Foundation for Statistical Computing).^39^ Separate models were estimated for men and women given that sex has been suggested to be an effect modifier of socioeconomic inequalities in all-cause mortality.^29^ To evaluate the differential vulnerability to lifestyle factors across educational attainment groups, we used Aalen’s additive hazard models to directly estimate additive interactions.^39,40^ Each lifestyle factor was evaluated one at a time while adjusting for covariates.

Causal mediation analyses using the marginal structural approach detailed by Lange et al^26,27,41^ were used to evaluate the differential exposure and vulnerability to lifestyle factors across educational attainment groups. The association between educational attainment and mortality was decomposed into 3 components: the mean pure direct effect, the mean pure indirect effect through each mediator (indicating differential exposure), and the mean effect of the mediated interaction between educational attainment and each mediator (indicating differential vulnerability). We fit an additive hazard model that included all lifestyle factors and covariates (listed above).

Four sensitivity analyses were conducted: (1) alcohol indexed by heavy episodic drinking, (2) stratified analyses by age group, (3) causal mediation for each lifestyle factor separately, and (4) analyses among all participants (ie, not stratified by sex). Results are presented in the eFigure and eTables 1 to 12 in the Supplement.

## Results

Participants included 415 764 adults (mean [SD] age, 49.4 [15.8] years; 55% women and 45% men; 17% Hispanic, 14% non-Hispanic Black, 64% non-Hispanic White, and 5% non-Hispanic other, of whom 12% were American Indian or Alaska Native, 53% Asian or Pacific Islander, and 35% other [including multiple race and ethnicity]), of whom 45% reported low educational attainment, 28% reported medium educational attainment, and 27% reported high educational attainment (Table 1). Participants were followed up for a mean (SD) of 8.8 (5.2) years during which 49 096 deaths were observed. At baseline, 58% of participants were category I drinkers (lowest drinking category), 55% had never smoked, 35% had a healthy weight, and 42% were physically active. Low educational attainment was associated with increased mortality, with 187.4 deaths per 10 000 person-years among those with low educational attainment compared with 105.7 deaths per 10 000 person-years among those with medium educational attainment and 69.8 deaths per 10 000 person-years among those with high educational attainment. Unhealthy lifestyle factors with respect to smoking, BMI (obesity), and physical inactivity were more prevalent among participants with lower educational attainment. The prevalence of category III drinking was largely similar across educational attainment groups, although category I drinking was more prevalent among higher educational attainment groups.

### Educational Attainment and Lifestyle Interactions

Table 2 presents the results of the additive interaction of educational attainment with each lifestyle factor, indicating differential vulnerability; these models did not adjust for other lifestyle factors. To improve clarity, we focused the presentation of results on the comparison between the groups with the lowest and highest educational attainment and lifestyle factors. Educational attainment was associated with all-cause mortality across all models; low educational attainment (compared with high educational attainment) was associated with 13.1 (95% CI, 9.2–16.9) to 96.0 (95% CI, 88.2–103.8) additional deaths per 10 000 person-years among individuals with the “best” lifestyle factor.

With respect to alcohol use, the highest drinking category (category III) was associated with increased mortality among men (independent of educational attainment) and women with low educational attainment. Among those with high educational attainment, the highest drinking category (compared with the lowest [category I]) was associated with increased mortality among men (107.8 [95% CI, 66.7–149.0] additional deaths per 10 000 person-years) but not women (22.0 [95% CI, −7.6 to 51.6] additional deaths per 10 000 person-years). However, among those with low educational attainment, the highest drinking category was associated with high mortality for both men (150.4 [95% CI, 131.5–169.3] additional deaths per 10 000 person-years) and women (133.9 [95% CI, 105.4–162.5] additional deaths per 10 000 person-years). In other words, we observed an additive interaction among women such that the presence of both low educational attainment and the highest drinking category (category III) resulted in 85.0 (95% CI, 43.8–126.3) additional deaths per 10 000 person-years than would have been expected from low educational attainment or category III drinking individually (ie, 133.9 − 22.0 − 27.0 = 84.9). We did not find evidence of additive interaction between drinking and educational attainment among men; that is, the presence of both low educational attainment and category III drinking did not result in more or fewer deaths than would be expected from low educational attainment or high-risk drinking individually.

The opposite pattern was observed for BMI, whereby an interaction was observed among men and not women. Among those with high educational attainment, obesity (compared with healthy weight) was associated with increased mortality among men (15.6 [95% CI, 7.9–23.4] additional deaths per 10 000 person-years) and women (7.0 [95% CI, 1.2–12.7] additional deaths per 10 000 person-years). The mortality rate associated with low educational attainment and obesity was high among men (81.6 [95% CI, 74.1–89.0] additional deaths per 10 000 person-years) and women (60.3 [95% CI, 55.0–65.5] additional deaths per 10 000 person-years). Notably, we observed an additive interaction among men, such that the presence of both low educational attainment and obesity resulted in 30.0 (95% CI, 18.5–41.5) fewer deaths per 10 000 person-years than would have been expected from low educational attainment or obesity individually (ie, 81.6 − 15.6 − 96.0 = −30.0). We did not find evidence of an additive interaction between educational attainment and obesity among women.

Lastly, regarding the association of educational attainment with smoking and physical activity, an additive interaction was observed for both men and women. Daily smoking (relative to never smoking) was associated with 26.2 (95% CI, 12.5–39.9) additional deaths per 10 000 person-years among men with low educational attainment than among men with high educational attainment, with a stronger association identified for women (48.2 [95% CI, 36.3–60.1] additional deaths per 10 000 person-years). Similarly, for physical activity, being sedentary (relative to active) was associated with 31.9 (95% CI, 21.4–42.3) additional deaths per 10 000 person-years among men with low educational attainment compared with among men with high educational attainment, with a similar association identified for women (34.1 [95% CI, 26.1–42.1] additional deaths per 10 000 person-years).

### Mediation Analysis

Table 3 presents the results of mediation analyses, simultaneously modeling all lifestyle factors and covariates. To improve clarity, we focused the presentation of results on the comparison between the low and high educational attainment groups. Among men, low educational attainment was associated with 83.6 (95% CI, 81.8–85.5) additional deaths per 10 000 person-years, of which 66% (95% CI, 63%−69%) were mediated by lifestyle factors. That is, if a hypothetical intervention brought the level of each lifestyle factor in the group with low educational attainment to the level seen in the group with high educational attainment (ie, improved lifestyle factors), a decrease of 66% of all-cause deaths would result among those with low educational attainment, indicating, in absolute terms, that 55.1 (95% CI, 53.2–57.0) fewer deaths per 10 000 person-years would occur. A similar pattern was observed for women, but the mortality rate associated with low educational attainment was smaller and the proportion mediated by lifestyle factors was greater. Specifically, low educational attainment was associated with 54.8 (95% CI, 53.4–56.2) additional deaths per 10 000 person-years, of which 80% (95% CI, 76%−83%) were mediated by lifestyle factors.

An additional novel finding was that socioeconomic inequalities in all-cause mortality (for both men and women) were primarily driven by the uneven distribution of lifestyle risk factors. That is, the differential vulnerability associated with each lifestyle factor discussed earlier was attenuated when accounting for differential exposure and other unhealthy lifestyle factors. More specifically, among men, 30% of the association between low educational attainment and mortality was mediated by smoking (differential exposure), 27% by physical activity, 16% by alcohol use, and 6% by BMI. The interaction between educational attainment and lifestyle factors (differential vulnerability) was relatively small and negative; a negative estimate indicates that the mortality rate associated with both low educational attainment and the lifestyle factors of the low educational attainment group was smaller (ie, was protective) compared with the sum of the mortality rate associated with having only low educational attainment or the lifestyle factors of the low educational attainment group. The results among women were similar to those of men with respect to differential exposure, whereby 24% of the association between low educational attainment and mortality was mediated by smoking, 23% by physical activity, and 11% by alcohol use. Body mass index was not a significant mediator. In contrast to men, the association between low educational attainment and mortality among women was attributable to differential vulnerability to alcohol use in 8% and physical activity in 10%. That is, we observed a greater mortality rate when both low educational attainment and the lifestyle factors of the low educational attainment group were present (ie, were more deleterious together) compared with the sum of the mortality rate when only low educational attainment or the lifestyle factors of the low educational attainment group were present.

## Discussion

The current study is novel in comprehensively evaluating multiple lifestyle factors and quantifying the magnitude and mechanisms through which lifestyle factors contribute to socioeconomic inequalities. Notably, lifestyle risk factors are themselves associated with structural and social determinants of health.^8^ The current results have important public health implications in that they identify subgroups and lifestyle risk factors as potential intervention targets that could yield important public health benefits, thus helping to inform priorities in the context of limited resources. In line with other studies,^2,21,42^ our results demonstrate that lower SES (operationalized as educational attainment) is associated with a higher prevalence of unhealthy lifestyle factors and all-cause mortality. Low SES was also associated with greater mortality among men, compared with women, in line with a previous study.^43^ In addition, we found that lifestyle factors explained 66% (men) and 80% (women) of the association between low SES and all-cause mortality. One study^29^ has previously used a similar comprehensive approach, finding that multiple lifestyle factors and comorbidities explained 36% of the association between low SES and all-cause mortality. Notable differences between that study and ours include the operationalization of SES (binary vs categorical [comparing the lowest and highest categories], respectively), the scale (multiplicative vs additive, respectively), the cultural context (predominantly European vs US, respectively), and modeling approaches (including a broader range of mediators vs focused on lifestyle factors only, respectively). Despite the difference in the proportion explained, the findings clearly suggest that public health interventions among groups with low SES have the potential to significantly increase their life expectancy and reduce socioeconomic inequalities in mortality by targeting lifestyle risk factors and the socioenvironmental context within which these risk factors develop.

Our results also provide some insight into the mechanisms underlying socioeconomic inequalities, suggesting they are largely driven by differential exposure as opposed to differential vulnerability, which has been hypothesized previously.^20,21,44^ Notably, given that lifestyle factors are not developed in isolation, the results may be taken more broadly to suggest that an important way in which socioeconomic inequalities in health may be produced is through differential exposure to the structural and social determinants of health, which drive the development and reinforcement of lifestyle risk factors.^8^ Taken a step further, these findings support previous calls for the US to adopt the best practices of other wealthy nations in providing communal assistance and preventive services throughout the life course.^45^

Little evidence for differential vulnerability was identified, with the exception of more deleterious effects of physical activity and alcohol use among women with low SES compared with high SES. This finding has not been previously described; past studies that focused on alcohol use have presented combined results for men and women^21,30,42^ or identified differential vulnerability among both Danish men and women.^46^ Studies using a comprehensive approach with causal mediation analyses have focused on cause-specific mortality, such as alcohol-attributable^30^ or cardiovascular-related mortality,^31^ and are not comparable with our results.

Lastly, with regard to each lifestyle factor, our results add to this literature by showing that socioeconomic inequalities in all-cause mortality were driven most by smoking and physical inactivity followed by alcohol use. The independent association with BMI was minimal; the apparent protective effect of BMI and SES from our interaction model for men largely disappeared when controlling for differential exposure and other lifestyle factors. Overall, these findings suggest that public health interventions that target smoking, physical inactivity, alcohol use, and the social, structural, and environmental contexts in which these behaviors develop among groups with low SES may yield important reductions in socioeconomic inequalities in mortality.

### Limitations

In interpreting the results presented above, a number of limitations should be considered, and causal interpretations should be avoided. First, causal mediation models have strong assumptions: no unmeasured confounders for the exposure-outcome, exposure-mediator, and mediator-outcome relationships as well as no mediator-outcome confounders caused by the exposure. In addition, mediators are assumed to have no causal effect on each other. The choice of covariates is important, and residual confounding by unmeasured risk factors (eg, adverse early-life events) is possible, which may have overestimated the association between educational attainment and mortality. The results of our sensitivity analyses, including (1) changing the operationalization of alcohol use, (2) stratifying by age groups, (3) evaluating mediators one at a time, and (4) not stratifying by sex, yielded results that aligned with our main analyses. Second, the data arose from participants’ self-report from a single time point. Accordingly, we have assumed that educational attainment precedes lifestyle risk factors; although educational attainment would have been achieved before participants’ report of the lifestyle factors, reverse causality (eg, other indexes of SES associated with educational attainment, leading to unhealthy lifestyles) is possible. In addition, reporting bias and changes in lifestyle factors over time may have introduced misclassification and underestimated the association between lifestyle factors and mortality. Third, we did not account for the complex survey design of the NHIS given the analytical and computational complexity of the analyses. Censoring is also an important consideration. Those who could not be matched with the NDI (5%) were right censored, which likely has little effect on our results. However, left censoring (experienced by those who died before survey onset or 25 years of age) may have been experienced to a greater degree by those with low SES and unhealthy lifestyle factors (particularly alcohol use^47^) and may have led to an underestimation of our results.

## Conclusions

Overall, the results presented above demonstrate that differential exposure to lifestyle risk factors may be an important driver of socioeconomic inequalities in health. Multilevel public health interventions that target smoking, physical activity, and alcohol use, along with the broader environmental and social determinants that can profoundly influence lifestyle behaviors, may yield the greatest benefits when targeting women and groups with low SES. Targeting lifestyle risk factors alone, without consideration of more fundamental forces, such as poverty, structural racism, and limited opportunity, will not likely improve socioeconomic inequalities. Future work should endeavor to understand these lifestyle mediators within the context of other mediators of SES inequalities that can operate across the life course to influence health and mortality, such as environmental quality, chronic and acute stressors, and access to health care.

## Supplementary Material

Supplemental Material Puka et al. 2022 **eMethods.** Supplementary Methods
**eFigure.** Survival Probabilities Stratified by Sex and Education
**eTable 1.** Characteristics at Baseline Among Participants With Complete and Missing Data
**eTable 2.** Heavy Episodic Drinking at Baseline, Stratified by Sex and Education
**eTable 3.** Results of Additive Hazard Models; Alcohol Use Indexed by Heavy Episodic Drinking
**eTable 4.** Results of Causal Mediation Analyses; Alcohol Use Indexed by Heavy Episodic Drinking
**eTable 5.** Results of Additive Hazard Models; Among Men and Stratified by Age
**eTable 6.** Results of Causal Mediation Analyses; Among Men and Stratified by Age
**eTable 7.** Results of Additive Hazard Models; Among Women and Stratified by Age
**eTable 8.** Results of Causal Mediation Analyses; Among Women and Stratified by Age
**eTable 9.** Results of Causal Mediation Analyses; Evaluating One Mediator at a Time
**eTable 10.** Participant Characteristics at Baseline, Stratified by Education
**eTable 11.** Results of Additive Hazard Models; Among All Participants
**eTable 12.** Results of Causal Mediation Analyses; Among All Participants

## Figures and Tables

**Figure.**
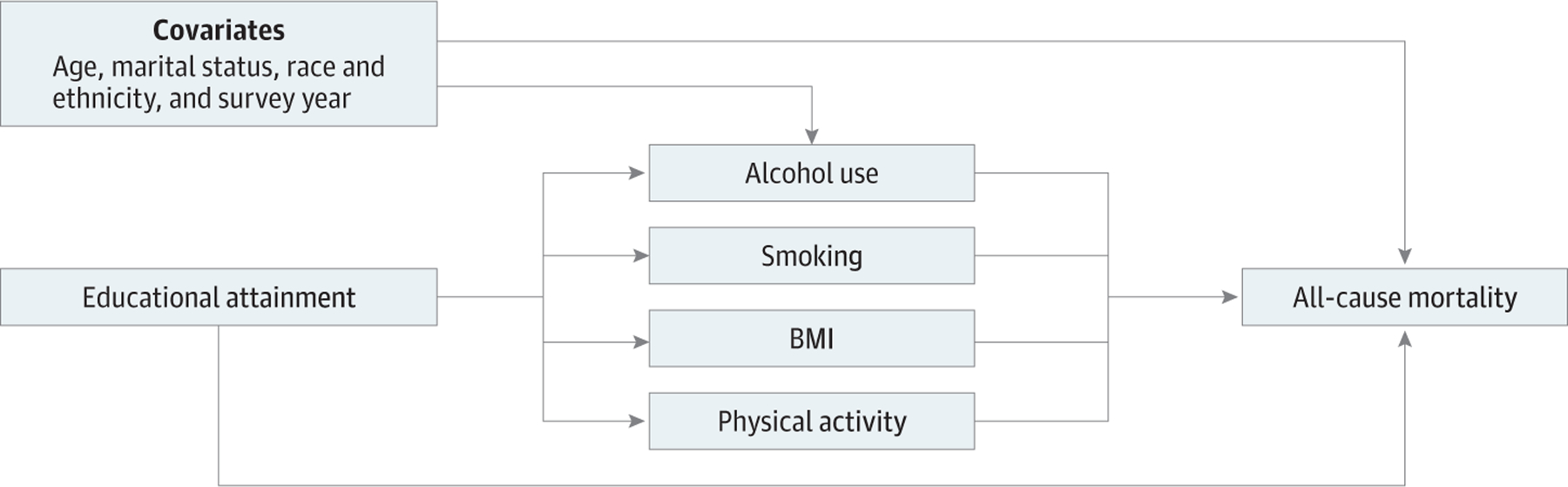
Diagram of the Association Among Socioeconomic Status, Lifestyle Risk Factors, Covariates, and All-Cause Mortality To improve clarity, arrows between the covariates and each lifestyle risk factor are not shown. BMI indicates body mass index.

**Table 1.** Participant Characteristics at Baseline, Stratified by Sex and Educational Attainment^a^

Characteristic	Complete sample (N = 415 764)	Men			Women		
		Low educational attainment (n = 83 531)	Medium educational attainment (n = 49 242)	High educational attainment (n = 52 997)	Low educational attainment (n = 105 314)	Medium educational attainment (n = 66 399)	High educational attainment (n = 58 281)
Age at baseline, mean (SD), y	49.4 (15.8)	50.4 (15.9)	47.4 (14.6)	47.8 (14.8)	53.0 (16.8)	47.9 (15.3)	46.2 (14.5)
Follow-up, mean (SD), y	8.8 (5.2)	8.7 (5.2)	8.7 (5.3)	8.7 (5.2)	9.1 (5.2)	8.9 (5.3)	8.7 (5.3)
No. of person-years	3 672 747	722 869	428 647	462 226	959 811	591 197	507 998
All-cause deaths, No. (%)	49 096 (12)	14 508 (17)	5306 (11)	4042 (8)	17 036 (16)	5471 (8)	2733 (5)
Death rate, per 10 000 person-years	133.7	200.7	123.8	87.4	177.5	92.5	53.8
Alcohol use, %							
Never drinker	31	26	19	17	51	32	24
Former drinker	7	12	8	5	6	5	4
Category I (lowest)	58	56	68	75	40	60	68
Category II	2	3	3	2	2	2	3
Category III (highest)	1	3	2	1	1	1	1
Smoking, %							
Never smoker	55	39	45	63	58	57	71
Former smoker	24	30	29	26	19	22	20
Current some-day smoker	4	6	5	4	4	4	3
Current everyday smoker	17	26	20	8	19	17	6
Body mass index, %							
Underweight	2	1	1	0	2	2	3
Healthy weight	35	27	26	33	34	39	53
Overweight	36	43	44	47	32	29	26
Obese	27	29	30	20	32	30	19
Physical activity, %							
Active	42	34	51	63	27	42	56
Somewhat active	18	14	16	17	18	22	21
Sedentary	40	51	33	20	55	36	23
Race and ethnicity, %							
Black, non-Hispanic	14	15	14	8	18	18	11
Hispanic	17	25	12	7	24	13	8
White, non-Hispanic	64	57	69	76	55	66	73
Other, non-Hispanic^b^	5	3	4	9	4	4	9
Married or cohabitating, %	56	60	59	65	48	51	58

a Educational attainment was categorized as low (high school diploma or less), medium (some college but no bachelor’s degree), or high (bachelor’s degree or more).

b Other, non-Hispanic is composed of 12% American Indian or Alaska Native, 53% Asian or Pacific Islander, and 35% other, including multiple race and ethnicity.

**Table 2.** Results of Additive Hazard Models Evaluating Additive Interaction of Education and Each Lifestyle Risk Factor on All-Cause Mortality^a^

Risk factor	Additional deaths per 10 000 person-years (95% CI)	
	Men	Women
Alcohol use × educational attainment		
High educational attainment, category I drinking	1 [Reference]	1 [Reference]
Low educational attainment, category I drinking	58.9 (54.4 to 63.4)	27.0 (23.3 to 30.7)
High educational attainment, category III drinking	107.8 (66.7 to 149.0)	22.0 (−7.6 to 51.6)
Low educational attainment, category III drinking	150.4 (131.5 to 169.3)	133.9 (105.4 to 162.5)
Additional deaths due to interaction	−16.2 (−61.2 to 29.1)	85.0 (43.8 to 126.3)
Smoking × educational attainment		
High educational attainment, never smoker	1 [Reference]	1 [Reference]
Low educational attainment, never smoker	31.2 (26.3 to 36.1)	13.1 (9.2 to 16.9)
High educational attainment, everyday smoker	81.0 (69.4 to 92.7)	51.9 (41.8 to 61.9)
Low educational attainment, everyday smoker	138.4 (131.9 to 144.9)	113.1 (107.3 to 118.9)
Additional deaths due to interaction	26.2 (12.5 to 39.9)	48.2 (36.3 to 60.1)
Body mass index × educational attainment		
High educational attainment, healthy weight	1 [Reference]	1 [Reference]
Low educational attainment, healthy weight	96.0 (88.2 to 103.8)	46.9 (42.1 to 51.7)
High educational attainment, obese	15.6 (7.9 to 23.4)	7.0 (1.2 to 12.7)
Low educational attainment, obese	81.6 (74.1 to 89.0)	60.3 (55.0 to 65.5)
Additional deaths due to interaction	−30.0 (−41.5 to −18.5)	6.5 (−2.0 to 14.9)
Physical activity × educational attainment		
High educational attainment, active	1 [Reference]	1 [Reference]
Low educational attainment, active	44.7 (39.6 to 49.8)	20.8 (16.5 to 25.1)
High educational attainment, sedentary	54.5 (46.3 to 62.7)	35.5 (29.6 to 41.4)
Low educational attainment, sedentary	131.1 (125.4 to 136.8)	90.3 (86 to 94.6)
Additional deaths due to interaction	31.9 (21.4 to 42.3)	34.1 (26.1 to 42.1)

a Separate models were conducted for each lifestyle factor, and all models were adjusted for age (as timescale), race and ethnicity, marital status, and survey year.

**Table 3.** Results of Causal Mediation Analyses Evaluating the Extent to Which the Association Between Education and All-Cause Mortality Was Mediated by Lifestyle Risk Factors^a^

**Risk factor**	**Men**		**Women**	
	**Additional deaths per 10 000 person-years (95% CI)**	**Proportion mediated (95% CI)** ^b^	**Additional deaths per 10 000 person-years (95% CI)**	**Proportion mediated (95% CI)** ^b^
Total effect of low educational attainment	83.6 (81.8 to 85.5)	100	54.8 (53.4 to 56.2)	100
Direct effect of low educational attainment	28.5 (26.6 to 30.4)	34 (32 to 36)	11.1 (9.7 to 12.5)	20 (18 to 23)
Indirect effect of low educational attainment	55.1 (53.2 to 57.0)	66 (63 to 69)	43.7 (42.2 to 45.3)	80 (76 to 83)
Alcohol use				
Differential exposure	13.2 (11.8 to 14.6)	16 (14 to 17)	6.1 (5.1 to 7.1)	11 (9 to 13)
Differential vulnerability	−5.0 (−6.6 to −3.3)	−6 (−8 to −4)	4.2 (2.9 to 5.4)	8 (5 to 10)
Smoking				
Differential exposure	25.5 (24.1 to 26.9)	30 (29 to 32)	12.9 (11.9 to 13.9)	24 (22 to 25)
Differential vulnerability	−2.6 (−4.2 to −0.9)	−3 (−5 to −1)	1.6 (0.3 to 2.8)	3 (1 to 5)
Body mass index				
Differential exposure	5.2 (3.9 to 6.6)	6 (5 to 8)	1.4 (0.4 to 2.4)	3 (1 to 4)
Differential vulnerability	−3.1 (−4.8 to −1.5)	−4 (−6 to −2)	−0.2 (−1.5 to 1.0)	0 (−3 to 2)
Physical activity				
Differential exposure	22.2 (20.8 to 23.6)	27 (25 to 28)	12.5 (11.5 to 13.5)	23 (21 to 24)
Differential vulnerability	−0.4 (−2.1 to 1.3)	0 (−2 to 1)	5.3 (4.1 to 6.6)	10 (7 to 12)

a The model was adjusted for age (as timescale), race and ethnicity, marital status, and survey year; for simplicity, only the effect of low educational attainment (compared with high educational attainment) is presented.

b Proportion mediated is the ratio between the effect and the total effect ×100.

## Key Points

### Question

To what extent can the association between socioeconomic status (SES) and mortality be explained by differential exposure to lifestyle factors (such that unhealthy lifestyle factors are more prevalent in groups with lower SES) and differential vulnerability to lifestyle factors (such that the same exposure to unhealthy lifestyle factors is associated with more deleterious outcomes in groups with lower SES)?

### Findings

In this nationwide cohort study of 415 764 US adults, a mediation analysis showed that lifestyle factors explained 66% (men) and 80% (women) of the association between educational attainment and all-cause mortality. Inequalities in mortality were primarily a result of greater exposure and clustering of unhealthy lifestyle factors among groups with lower educational attainment; with some exception, there was little evidence for differential vulnerability to lifestyle factors.

### Meaning

Public health interventions to create equality in the socioenvironmental contexts that shape lifestyle factors and to reduce exposure to lifestyle risk factors among groups with low SES have the potential to significantly increase life expectancy and reduce socioeconomic inequalities in mortality.
