# Supplementary material for: Educational Attainment and Lifestyle Risk Factors Associated With All-Cause Mortality in the US
Source: JAMA Health Forum. Author manuscript; Available in PMC 2022 Apr 19. (PMC8994133; doi:10.1001/jamahealthforum.2022.0401)
Supplement: Supplemental Material Puka et al. 2022 — eMethods. Supplementary Methods eFigure. Survival Probabilities Stratified by Sex and Education eTable 1. Characteristics at Baseline Among Participants With Complete and Missing Data eTable 2. Heavy Episodic Drinking at Baseline, Stratified by Sex and Education eTable 3. Results of Additive Hazard Models; Alcohol Use Indexed by Heavy Episodic Drinking eTable 4. Results of Causal Mediation Analyses; Alcohol Use Indexed by Heavy Episodic Drinking eTable 5. Results of Additive Hazard Models; Among Men and Stratified by Age eTable 6. Results of Causal Mediation Analyses; Among Men and Stratified by Age eTable 7. Results of Additive Hazard Models; Among Women and Stratified by Age eTable 8. Results of Causal Mediation Analyses; Among Women and Stratified by Age eTable 9. Results of Causal Mediation Analyses; Evaluating One Mediator at a Time eTable 10. Participant Characteristics at Baseline, Stratified by Education eTable 11. Results of Additive Hazard Models; Among All Participants eTable 12. Results of Causal Mediation Analyses; Among All Participants [file NIHMS1797399-supplement-Supplemental_Material_Puka_et_al__2022.pdf]

Supplementary Online Content

Puka K, Buckley C, Mulia N, Lasserre AM, Rehm J, Probst C. Educational attainment and lifestyle risk factors associated with all-cause mortality in the US. *JAMA Health Forum.* 2022;3(4):e220401. doi:10.1001/jamahealthforum.2022.0401

eMethods. Supplementary Methods
eFigure. Survival Probabilities Stratified by Sex and Education
eTable 1. Characteristics at Baseline among participants with complete and missing data.
eTable 2. Heavy Episodic Drinking at Baseline, Stratified by Sex and Education
eTable 3. Results of Additive Hazard Models; Alcohol Use Indexed by Heavy Episodic Drinking
eTable 4. Results of Causal Mediation Analyses; Alcohol Use Indexed by Heavy Episodic Drinking
eTable 5. Results of Additive Hazard Models; Among Men and Stratified by Age
eTable 6. Results of Causal Mediation Analyses; Among Men and Stratified by Age
eTable 7. Results of Additive Hazard Models; Among Women and Stratified by Age
eTable 8. Results of Causal Mediation Analyses; Among Women and Stratified by Age
eTable 9. Results of Causal Mediation Analyses; Evaluating One Mediator at a Time
eTable 10. Participant Characteristics at Baseline, Stratified by Education
eTable 11. Results of Additive Hazard Models; Among All Participants
eTable 12. Results of Causal Mediation Analyses; Among All Participants

This supplementary material has been provided by the authors to give readers additional information about their work.

© 2022 Puka K et al. *JAMA Health Forum.*

eMethods. Supplementary Methods

Data came from the United States (US) National Health Interview Survey (NHIS). NHIS utilized a complex, multistage sample design that involved stratification, clustering, and oversampling of specific population subgroups. Every year approximately 35,000 households are enrolled, from which one adult is randomly selected for a face-to-face interview. An annual assessment of alcohol use in sufficient detail started in 1997, and NHIS data up to 2014 have been linked to the National Death Index (NDI). Therefore, this study included pooled NHIS data from 1997 to 2014. The NDI contains information on vital status, time of death, and time last presumed alive with follow-up to December 31, 2015. Our sample was comprised of the adults randomly selected for the face-to-face interview, with no missing data on our exposure, mediators, outcome, or covariates. Of the 552,837 individuals who participated in NHIS, 69,665 (31%) were younger than 25 years or older than 85 years and were not eligible for our study. Of the 483,172 eligible participants, 23,474 (5%) could not be matched with the NDI and 43,934 (9%) were missing data on the variables used in our analyses (specifically, lifestyle factors, age, sex, race/ethnicity, and/or marital status). The final sample size was 415,764. Of the eligible participants, those with complete and missing data were largely similar across a range of characteristics (eTable 1).

Alcohol use was categorized into mutually exclusive categories based on the average grams of pure alcohol consumed per day. Participants were asked to report 1) whether they have consumed at least 12 drinks of any type of alcoholic beverage in a) in any one year and b) over their entire life, 2) the number of days, in the past 12 months, that they had any type of alcoholic beverage and the average number of standard drinks consumed on those days, and 3) the number of heavy drinking days in the past year, defined as days on which they had 5 or more standard drinks on one day. These questions were consistent across all survey years (1997-2014), with the exception of heavy drinking, which in 2014 was modified such that heavy drinking for women was defined as 4 (rather than 5) or more drinks per day. These questions were used to calculate the average consumption of pure alcohol per day, assuming 14 grams of pure alcohol per standard drink. The number of heavy drinking days (conservatively assuming 5 drinks per day) was added to the average consumption per day among participants reporting an

© 2022 Puka K et al. *JAMA Health Forum.*

average of less than 5 drinks per drinking day. Alcohol use was categorized as 1) never drinkers (no drinks in the past year and less than 12 drinks in any one year or entire life), 2) former drinkers (no drinks in the past year but have had at least 12 drinks in any one year), 3) category I (men: up to 20 (women) or 40 (men) grams per day; women: up to 20 grams per day), 4) category II (men:21-40 (women) or 41-60 (men) grams per day; women: 21-40 grams per day), 5) category III (men: ≥41 (women) or ≥61 (men) grams per day; women: ≥41 grams per day). Assuming 14 grams of pure alcohol per standard drink, these categories of alcohol use are equivalent to: category I (up to 10 (women) or 20 (men) drinks per week), category II (10-20 (women) or 20-30 (men) drinks per week), and category III (>20 (women) or >30 (men) drinks per week). Category I drinking behavior was used as the reference category, given that this group comprised of the majority of the population and because *never drinkers* may have poorer health outcomes.[1] Lastly, as a sensitivity analysis, alcohol use was indexed using heavy episodic drinking (HED) based on the number of heavy drinking days (≥5 drinks a day) in the past 12 months. This indicator is not a measure of "binge drinking," generally defined as 5+ drinks (sometimes 4+ for women) on a single drinking occasion. HED was categorized into four categories based on the number of heavy drinking days in the past 12 months: no HED (0 heavy drinking days), HED less than once a month (1-11 heavy drinking days), HED at least once a month but less than once a week (12-51 heavy drinking days), and HED once a week or more (52-365 heavy drinking days).

With respect to smoking, participants were asked to report whether they 1) have smoked at least 100 cigarettes over their entire life, and 2) whether they currently smoke. Smoking was categorized as never smokers (reference category), former smokers, current some day smokers, and current everyday smokers.

With respect to physical activity, participants were asked to report 1) how often they performed a) vigorous or b) light-moderate leisure-time physical activities of at least 10 minutes that caused a) heavy sweating or large increases in breathing or heart rate or b) only light sweating or a slight to moderate increase in breathing or heart rate, and 2) how long they do those a) vigorous or b) light or moderate leisure-time physical activities each time. No timeframe (e.g., over the past year, or past month) was specified for either question. The length

of physical activity per week was calculated and combined, assuming that 1 minute of vigorous physical activity is equivalent to 2 minutes of moderate physical activity [2]. Physical activity was categorized using WHO recommendations of 150-300 minutes of moderate-intensity physical activity per week [3].

Statistical Analyses

To evaluate the interaction (joint effects) of education and lifestyle risk factors on mortality (Objective 1), Aalen's additive hazard models were used to directly estimate additive interaction [4,5]. Additive interaction was the focus given that it is of greater importance for public health [4]. An additional benefit is that the coefficients obtained from Aalen models are collapsible, unlike those obtained from Cox models;[6] that is, one can meaningfully compare parameter estimates from Aalen models with different covariates, but should not do so for Cox models since the addition of a covariate could shift the baseline hazard rather than simply altering the slops of the hazard function. The hazard of all-cause mortality for person *i* at age *t* was modeled as a linear function of the exposure (E; education), the lifestyle risk factor (M), their interaction (E x M), covariates (C), and an unspecified baseline hazard (λ_0):

$$\lambda_i(t) = \lambda_0(t) + \alpha_1 E_i + \alpha_2 M_i + \alpha_3 (E\ x\ M)_i + \beta(t) C_i$$

With regard to interpretation, α_3 directly estimates the number of additional events per person year at risk due to their additive interaction. This semiparametric model is flexible and can incorporate time-varying covariate effects (i.e., β(t), where the effect of the covariate is not constant over time). Each lifestyle risk factor was evaluated one at a time, and models were adjusted for age (used as the time scale), and the following categorical variables: race/ethnicity, marital status, and survey year. Separate models were estimated for men and women given that sex has been suggested to be an effect modifier of socioeconomic inequalities on all-cause mortality [7]. The graphical techniques and tests described by Scheike and Martinussen [5] suggested that race/ethnicity, smoking, and physical activity should be modeled as age-varying effects; this is equivalent to a violating the proportional hazards assumption in Cox models. Aalen models are flexible and the effects of race/ethnicity were included in the model as age-

© 2022 Puka K et al. *JAMA Health Forum.*

varying. Sensitivity analyses by age subgroups (where the age-invariant assumption was met) were used to examine the impact of modeling smoking and physical activity as age-invariant.

The marginal structural approach described by Lange et al. [8-10] was used to evaluate the extent to which lifestyle risk factors mediated the relationship between education and mortality (Objective 2). Briefly, this flexible approach uses a counterfactual framework and allows for the direct parameterization of natural direct and indirect effects, multiple mediators, and exposure-mediator interactions. The total effect of education on mortality was decomposed into three components: the average pure direct effect, the average pure indirect effect through each mediator (indicating differential exposure), and the average effect of the mediated interaction between education and each mediator (indicating differential vulnerability). The proportion of the total effect mediated by each lifestyle risk factor was also calculated. We fit an additive hazard model including all lifestyle risk factors (alcohol use, smoking, BMI, physical activity) and covariates (age [used as the time scale], race/ethnicity, marital status, and survey year), and fit separate models for men and women. Robust standard errors were not used given the size of the sample and computational limitations, despite the fact that the analyses were conducted on a specialized computing cluster.[5]

All analyses were completed in R 3.6.3, using the *timereg* package (version 1.9.8)[5]. The *timereg* package does not allow for complex sampling designs and survey weights were not utilized.

Statistical Code

The statistical code for this manuscript is publicly available at
https://github.com/kpuka/SIMAH_clean/tree/main/Puka_2022_SES_x_Lifestyles

Sensitivity Analyses

As sensitivity analyses, the analyses described above were repeated with small modifications. First, alcohol use was indexed using heavy episodic drinking (HED) based on the number of heavy drinking days (≥5 drinks a day) in the past 12 months (eTables 2 to 4). Second, analyses were stratified analyses by age group, to evaluate the impact of modeling smoking and physical

© 2022 Puka K et al. *JAMA Health Forum.*

activity as age-invariant (see the Statistical Analyses section above; eTables 5 to 8). The age categories of 25-59 years, 60-69 years, and 70-85 years, where the age-invariant assumption was met. Third, the causal mediation analyses (Objective 2) were conducing using one lifestyle factor at a time as the mediator, to address the assumption that mediators have no causal effect on each other (eTable 9). In these models, the other lifestyle factors were included as covariates. Fourth, analyses were repeated among all participants (i.e., not stratified by sex; eTables 10 to 12).

eReferences

1. Ng Fat L, Shelton N. Associations between self-reported illness and non-drinking in young adults. *Addiction* 2012;107:1612-20.

2. U.S. Department of Health Human Services. Physical Activity Guidelines for Americans (2nd edition). Washington, DC: U.S. Department of Health Human Services; 2018.

3. Bull FC, Al-Ansari SS, Biddle S, et al. World Health Organization 2020 guidelines on physical activity and sedentary behaviour. *British Journal of Sports Medicine* 2020;54:1451.

4. Rod NH, Lange T, Andersen I, Marott JL, Diderichsen F. Additive Interaction in Survival Analysis: Use of the Additive Hazards Model. *Epidemiology* 2012;23.

5. Scheike TH, Martinussen T. Dynamic Regression models for survival data: Springer, NY.; 2006.

6. Martinussen T, Vansteelandt S. On collapsibility and confounding bias in Cox and Aalen regression models. *Lifetime Data Anal* 2013;19:279-96.

7. Laine JE, Baltar VT, Stringhini S, et al. Reducing socio-economic inequalities in all-cause mortality: a counterfactual mediation approach. *Int J Epidemiol* 2020;49:497-510.

8. Lange T, Rasmussen M, Thygesen LC. Assessing Natural Direct and Indirect Effects Through Multiple Pathways. *American Journal of Epidemiology* 2013;179:513-8.

9. Lange T, Vansteelandt S, Bekaert M. A Simple Unified Approach for Estimating Natural Direct and Indirect Effects. *American Journal of Epidemiology* 2012;176:190-5.

10. Lange T, Hansen JV. Direct and indirect effects in a survival context. *Epidemiology* 2011;22:575-81.

© 2022 Puka K et al. *JAMA Health Forum.*

eFigure. Survival Probabilities Stratified by Sex and Education

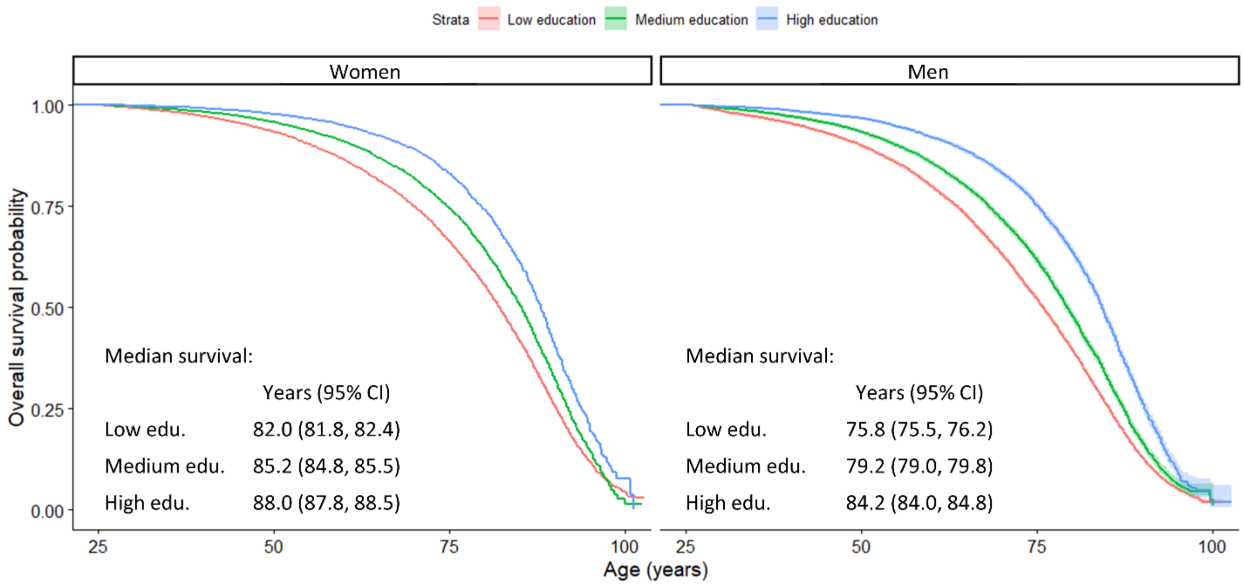

© 2022 Puka K et al. *JAMA Health Forum.*

eTable 1. Characteristics at Baseline among participants with complete and missing data.

	Men				Women		
	Completed follow-up	Loss to follow-up	SMD		Completed follow-up	Loss to follow-up	SMD
Sample size, n	185,770	26,920			229,994	40,488	
Age at baseline, mean yrs (SD)	48.9 (15.3)	47.8 (15.1)	0.069		49.8 (16.1)	49.7 (15.7)	0.007
Educational attainment			0.098				0.060
Low	45	50			46	49	
Medium	27	25			29	28	
High	29	25			25	23	
Alcohol use, %			0.124				0.131
Never drinker	21	26			38	45	
Former drinker	9	9			6	6	
Category I (lowest)	65	60			53	48	
Category II	2	2			2	2	
Category III (highest)	2	2			1	1	
Smoking, %			0.069				0.042
Never smoker	48	49			61	63	
Former smoker	28	25			20	19	
Current some day smoker	5	5			4	3	
Current everyday smoker	19	20			15	15	
BMI, %			0.126				0.153
Underweight	1	1			2	3	
Healthy weight	28	32			40	46	
Overweight	44	45			29	29	
Obese	27	22			28	22	
Physical activity, %			0.299				0.193
Active	47	36			39	32	
Somewhat active	16	12			20	17	
Sedentary	37	52			41	51	
Ethnicity, %			0.062				0.026
Black, non-Hispanic	13	13			16	16	
Hispanic	16	18			17	16	
White, non-Hispanic	66	63			62	63	
Other, non-Hispanic	5	5			5	5	
Married/cohabitating, %	61	59	0.045		51	52	0.012

SD: standard deviation; yrs: years.

SMD: standardized mean difference, with values of 0.2, 0.5, and 0.8 interpretable as small, medium, and large effect sizes, respectively.

© 2022 Puka K et al. *JAMA Health Forum.*

eTable 2. Heavy episodic drinking at Baseline, Stratified by Sex and Education

	Complete sample		Men				Women		
			Low education	Medium education	High education		Low education	Medium education	High education
Sample size	415,764		83,531	49,242	52,997		105,314	66,399	58,281
Heavy episodic drinking in past 12 months, %									
No HED	80		72	67	70		91	87	85
HED < 1 / month	11		12	17	18		5	9	11
HED < 1 / week	4		7	7	7		2	2	2
HED ≥ 1 / week	5		10	8	5		2	2	1

HED: heavy episodic drinking (≥5 drinks a day).

© 2022 Puka K et al. *JAMA Health Forum.*

eTable 3. Results of Additive Hazard Models; Alcohol Use Indexed By Heavy Episodic Drinking

	Men	Women
	Additional deaths per 10,000 person-years (95% CI)	Additional deaths per 10,000 person-years (95% CI)
**HED x Education**		
High education & no HED	Reference	reference
Low education & no HED	86.4 (80.9, 91.9)	51.9 (48.3, 55.5)
High education & HED ≥1/week	42.0 (31.4, 52.7)	5.8 (-5.6, 17.1)
Low education & HED ≥1/week	96.3 (87.7, 104.9)	75.5 (61.2, 89.9)
Extra deaths due to interaction	**-32.1 (-45.8, -18.5)**	**17.8 (-0.5, 36.1)**

All models are adjusted for age (as timescale), race/ethnicity, marital status, and survey year.
CI: confidence interval.

© 2022 Puka K et al. *JAMA Health Forum.*

eTable 4. Results of Causal Mediation Analyses,; Alcohol Use Indexed By Heavy Episodic Drinking.

	Men			Women	
	Additional deaths per 10,000 person-years (95% CI)	Proportion mediated (95% CI) [a]		Additional deaths per 10,000 person-years (95% CI)	Proportion mediated (95% CI) [a]
Total effect of low education	83.6 (81.9, 85.3)	100		53.8 (52.5, 55.1)	100
Direct effect of low education	34.3 (32.5, 36.1)	41 (39, 43)		19.9 (18.5, 21.3)	37 (35, 39)
Indirect effect of low education	49.3 (47.4, 51.1)	59 (56, 61)		33.9 (32.4, 35.3)	63 (60, 66)
HED: differential exposure	5.2 (4, 6.4)	6 (5, 8)		1.1 (0.2, 2)	2 (0, 4)
HED: differential vulnerability	-4.1 (-5.6, -2.5)	-5 (-7, -3)		-0.7 (-1.9, 0.4)	-1 (-3, 1)
Smoking: differential exposure	23.8 (22.6, 25)	29 (27, 30)		12.7 (11.8, 13.6)	24 (22, 25)
Smoking: differential vulnerability	-0.8 (-2.3, 0.7)	-1 (-3, 1)		1.4 (0.2, 2.6)	3 (0, 5)
BMI: differential exposure	5.3 (4.1, 6.5)	6 (5, 8)		1.3 (0.4, 2.3)	2 (1, 4)
BMI: differential vulnerability	-3.1 (-4.6, -1.5)	-4 (-5, -2)		0 (-1.2, 1.2)	0 (-2, 2)
Physical activity: differential exposure	21 (19.8, 22.2)	25 (24, 26)		12.2 (11.3, 13.1)	23 (21, 24)
Physical activity: differential vulnerability	1.9 (0.4, 3.4)	2 (0, 4)		5.9 (4.7, 7.1)	11 (9, 13)

The model adjusted for age (as timescale), race/ethnicity, marital status, and survey year; for simplicity, only the effect of low education (relative to high education) is presented. CI: confidence interval.
[a] Proportion mediated is the ratio between the effect and the total effect x 100

© 2022 Puka K et al. *JAMA Health Forum.*

eTable 5. Results of Additive Hazard Models; Among Men and Stratified by Age

Men	Aged 25-59 years	Aged 60-69 years	Aged 70-85 years
	Additional deaths per 10,000 person-years (95% CI)	Additional deaths per 10,000 person-years (95% CI)	Additional deaths per 10,000 person-years (95% CI)
**Alcohol Use x Education**			
High education, Category I drinking	Reference	Reference	Reference
Low education, Category I drinking	32.6 (29.3, 35.9)	103.6 (89.7, 117.5)	161.6 (135.2, 188.0)
High education, Category III drinking	48.7 (14.0, 83.5)	137.3 (39.6, 235.0)	394.1 (181.3, 606.9)
Low education, Category III drinking	84.1 (67.7, 100.6)	299.4 (234.7, 364.1)	447.3 (330.5, 564.1)
Additional deaths due interaction	2.8 (-35.6, 41.3)	58.5 (-59.1, 176.0)	-108.7 (-351.5, 134.0)
**Smoking x Education**			
High education, never smoker	Reference	Reference	Reference
Low education, never smoker	24.8 (21.1, 28.5)	60.4 (45.3, 75.5)	107.1 (78.0, 136.3)
High education, everyday smoker	30.7 (21.4, 40.0)	138.5 (103.7, 173.2)	525.0 (418.9, 631.2)
Low education, everyday smoker	62.5 (57.3, 67.7)	294.1 (270.3, 317.8)	631.3 (578.6, 684)
Additional deaths due interaction	7.0 (-4.0, 18.0)	**95.2 (52.2, 138.2)**	-0.8 (-119.0, 117.3)
**Body Mass Index x Education**			
High education, healthy weight	Reference	Reference	Reference
Low education, healthy weight	44.7 (39.3, 50.2)	171.9 (148.1, 195.8)	246.6 (206.7, 286.5)
High education, obese	4.8 (-0.6, 10.2)	23.6 (3.0, 44.3)	51.7 (2.6, 100.7)
Low education, obese	45 (39.5, 50.6)	129.9 (109, 150.7)	196 (156.3, 235.8)
Additional deaths due interaction	-4.5 (-13.0, 4.0)	**-65.7 (-98.2, -33.2)**	**-102.2 (-164.4, -39.9)**
**Physical Activity x Education**			
High education, active	Reference	Reference	Reference
Low education, active	27.6 (23.8, 31.4)	84.4 (68.8, 100.0)	103.9 (76.6, 131.2)
High education, sedentary	20.8 (15.1, 26.6)	83.5 (63.6, 103.4)	188.9 (148.6, 229.2)
Low education, sedentary	59.1 (54.8, 63.5)	193.8 (178.4, 209.3)	380.9 (352.9, 408.9)
Additional deaths due interaction	**10.7 (3.1, 18.3)**	**26.0 (-1.1, 53.1)**	**88.1 (38.6, 137.7)**

All models are adjusted for age (as timescale), race/ethnicity, marital status, and survey year. Bolded text highlights significant interactions.CI: confidence interval.

© 2022 Puka K et al. *JAMA Health Forum.*

eTable 6. Results of Causal Mediation Analyses; Among Men and Stratified by Age

**Men**	Ages 25-59 years			Ages 60-69 years			Ages 70-85 years	
	Additional deaths per 10,000 person-years (95% CI)	Proportion mediated (95% CI) [a]		Additional deaths per 10,000 person-years (95% CI)	Proportion mediated (95% CI) [a]		Additional deaths per 10,000 person-years (95% CI)	Proportion mediated (95% CI) [a]
Total effect of low education	38.9 (37.5, 40.3)	100		124.9 (119.9, 130)	100		202.1 (192.7, 211.5)	100
Direct effect of low education	18.4 (17, 19.8)	47 (44, 51)		41.4 (36.3, 46.5)	33 (30, 37)		44.7 (35.2, 54.2)	22 (18, 26)
Indirect effect of low education	20.4 (19.1, 21.7)	53 (49, 56)		83.5 (78.7, 88.3)	67 (62, 72)		157.4 (149.6, 165.2)	78 (73, 83)
Alcohol use: differential exposure	5.6 (4.4, 6.7)	14 (11, 17)		12 (8.1, 15.9)	10 (6, 13)		40.2 (32.3, 48.1)	20 (16, 23)
Alcohol use: differential vulnerability	-2.3 (-3.6, -1)	-6 (-9, -3)		0.7 (-3.9, 5.3)	1 (-3, 4)		-16.4 (-25.2, -7.6)	-8 (-12, -4)
Smoking: differential exposure	10.9 (9.8, 12)	28 (25, 31)		33.7 (29.8, 37.6)	27 (24, 30)		94.2 (86.3, 102)	47 (43, 50)
Smoking: differential vulnerability	-2.0 (-3.3, -0.7)	-5 (-8, -2)		9.0 (4.5, 13.5)	7 (4, 11)		-35.7 (-44.5, -26.9)	-18 (-22, -14)
BMI: differential exposure	0.7 (-0.4, 1.9)	2 (-1, 5)		3.0 (-0.9, 7)	2 (-1, 6)		13.5 (5.7, 21.3)	7 (3, 10)
BMI: differential vulnerability	0.1 (-1.3, 1.4)	0 (-3, 4)		-0.5 (-5.2, 4.1)	0 (-4, 3)		-7.4 (-16.1, 1.3)	-4 (-8, 1)
Physical activity: differential exposure	8.3 (7.1, 9.4)	21 (19, 24)		28.3 (24.3, 32.3)	23 (20, 26)		72.4 (64.4, 80.4)	36 (32, 40)
Physical activity: differential vulnerability	-0.8 (-2.1, 0.5)	-2 (-5, 1)		-2.6 (-7.3, 2)	-2 (-6, 2)		-3.4 (-12.2, 5.5)	-2 (-6, 3)

The model adjusted for age (as timescale), race/ethnicity, marital status, and survey year; for simplicity, only the effect of low education (relative to high education) is presented. CI: confidence interval.

[a] Proportion mediated is the ratio between the effect and the total effect x 100

© 2022 Puka K et al. *JAMA Health Forum.*

eTable 7. Results of Additive Hazard Models ; Among Women and Stratified by Age

Women	Aged 25-59 years	Aged 60-69 years	Aged 70-85 years
	Additional deaths per 10,000 person-years (95% CI)	Additional deaths per 10,000 person-years (95% CI)	Additional deaths per 10,000 person-years (95% CI)
**Alcohol Use x Education**			
High education, Category I drinking	Reference	Reference	Reference
Low education, Category I drinking	21.5 (18.8, 24.2)	43.0 (32.8, 53.2)	85.4 (64.1, 106.8)
High education, Category III drinking	14.5 (-9.7, 38.7)	17.5 (-51.1, 86.1)	146.0 (-28.8, 320.8)
Low education, Category III drinking	83.6 (58.4, 108.8)	188.1 (101.3, 274.9)	398.7 (238.8, 558.6)
Additional deaths due interaction	**47.6 (12.6, 82.6)**	**127.6 (16.9, 238.3)**	167.0 (-69.7, 403.7)
**Smoking x Education**			
High education, never smoker	Reference	Reference	Reference
Low education, never smoker	19.3 (16.7, 22.0)	43.4 (33.9, 52.9)	72.8 (54.5, 91.2)
High education, everyday smoker	14.0 (7.0, 21.0)	88.6 (57.3, 120.0)	350.6 (262.8, 438.4)
Low education, everyday smoker	48.8 (44.4, 53.2)	182.7 (164.3, 201.2)	478.5 (439.9, 517.1)
Additional deaths due interaction	**15.5 (7.0, 23.9)**	**50.7 (14.3, 87.1)**	55.2 (-39.8, 150.2)
**Body Mass Index x Education**			
High education, healthy weight	Reference	Reference	Reference
Low education, healthy weight	26.9 (23.7, 30.2)	73.2 (60.4, 86.0)	134.3 (110.1, 158.4)
High education, obese	5.7 (1.4, 9.9)	17.0 (1.2, 32.8)	23.9 (-13.6, 61.3)
Low education, obese	34.3 (30.4, 38.2)	90.7 (77.3, 104)	155.1 (129.5, 180.6)
Additional deaths due interaction	1.8 (-4.4, 7.9)	0.4 (-21.0, 21.8)	-3.2 (-46.3, 39.9)
**Physical Activity x Education**			
High education, active	Reference	Reference	Reference
Low education, active	21.8 (18.5, 25.0)	42.8 (31.8, 53.7)	58.6 (36.9, 80.2)
High education, sedentary	12.8 (9.0, 16.7)	62.5 (46.2, 78.8)	160.0 (126.0, 194.0)
Low education, sedentary	37.8 (34.6, 41)	128.3 (117.1, 139.4)	256.3 (235.4, 277.3)
Additional deaths due interaction	3.2 (-2.3, 8.7)	**23.0 (2.6, 43.5)**	**37.7 (-0.8, 76.3)**

All models are adjusted for age (as timescale), race/ethnicity, marital status, and survey year. Bolded text highlights significant interactions. CI: confidence interval.

© 2022 Puka K et al. *JAMA Health Forum.*

eTable 8. Results of Causal Mediation Analyses; Among Women and Stratified by Age

**Women**	Ages 25-59			Ages 60-69			Ages 70-85	
	Additional deaths per 10,000 person-years (95% CI)	Proportion mediated (95% CI) [a]		Additional deaths per 10,000 person-years (95% CI)	Proportion mediated (95% CI) [a]		Additional deaths per 10,000 person-years (95% CI)	Proportion mediated (95% CI) [a]
Total effect of low education	27.2 (26.2, 28.2)	100		73.2 (69.5, 77.0)	100		124.4 (116.7, 132.0)	100
Direct effect of low education	14.8 (13.8, 15.8)	54 (51, 58)		20.4 (16.7, 24.1)	28 (23, 32)		19.4 (11.7, 27.1)	16 (10, 21)
Indirect effect of low education	12.5 (11.4, 13.5)	46 (42, 50)		52.9 (49.7, 56.0)	72 (67, 78)		105.0 (100.2, 109.7)	84 (78, 91)
Alcohol use: differential exposure	2.5 (1.8, 3.2)	9 (7, 12)		8.0 (4.9, 11.1)	11 (7, 15)		16.6 (9.8, 23.4)	13 (8, 18)
Alcohol use: differential vulnerability	0.4 (-0.4, 1.3)	2 (-2, 5)		5.7 (2.2, 9.1)	8 (3, 13)		3.9 (-3.3, 11.1)	3 (-3, 9)
Smoking: differential exposure	3.6 (2.9, 4.3)	13 (11, 16)		20.8 (17.8, 23.8)	28 (25, 32)		64.6 (57.9, 71.3)	52 (47, 57)
Smoking: differential vulnerability	2.2 (1.3, 3.0)	8 (5, 11)		-2.0 (-5.4, 1.4)	-3 (-7, 2)		-31.9 (-39.0, -24.8)	-26 (-31, -20)
BMI: differential exposure	0.8 (0.1, 1.5)	3 (0, 5)		-0.1 (-3.2, 2.9)	0 (-4, 4)		0.0 (-6.7, 6.8)	0 (-6, 5)
BMI: differential vulnerability	-0.2 (-1.0, 0.7)	-1 (-4, 3)		1.5 (-1.9, 4.9)	2 (-3, 7)		1.5 (-5.6, 8.6)	1 (-5, 7)
Physical activity: differential exposure	4.2 (3.5, 4.9)	15 (13, 18)		16.7 (13.6, 19.8)	23 (19, 27)		52.2 (45.4, 59)	42 (37, 47)
Physical activity: differential vulnerability	-1.0 (-1.9, -0.1)	-4 (-7, -1)		2.4 (-1.1, 5.8)	3 (-1, 8)		-1.9 (-9.1, 5.3)	-2 (-7, 4)

The model adjusted for age (as timescale), race/ethnicity, marital status, and survey year; for simplicity, only the effect of low education (relative to high education) is presented.CI: confidence interval.
[a] Proportion mediated is the ratio between the effect and the total effect x 100

© 2022 Puka K et al. *JAMA Health Forum.*

eTable 9. Results of Causal Mediation Analyses; Evaluating One Mediator at a Time

	Men			Women	
**Alcohol use**	Additional deaths per 10,000 person-years (95% CI)	Proportion mediated (95% CI) [a]		Additional deaths per 10,000 person-years (95% CI)	Proportion mediated (95% CI) [a]
Total effect of low education	39.5 (35.3, 43.8)	100		23.2 (19.9, 26.5)	100
Direct effect of low education	32.0 (27.7, 36.3)	81 (71, 92)		12.9 (9.6, 16.3)	56 (42, 69)
Indirect effect of low education	7.6 (3.1, 12.0)	19 (8, 31)		10.3 (6.6, 14.0)	44 (28, 63)
Alcohol use: differential exposure	7.6 (3.8, 11.5)	19 (10, 28)		4.4 (1.5, 7.3)	19 (7, 31)
Alcohol use: differential vulnerability	-0.1 (-6.0, 5.8)	0 (-16, 14)		5.9 (1.3, 10.6)	26 (6, 45)
**Smoking**					
Total effect of low education	50.5 (46.3, 54.7)	100		32.2 (28.9, 35.5)	100
Direct effect of low education	27.5 (23.2, 31.8)	54 (47, 62)		15.1 (11.7, 18.5)	47 (37, 57)
Indirect effect of low education	23.0 (18.5, 27.6)	46 (36, 56)		17.1 (13.4, 20.8)	53 (41, 66)
Smoking: differential exposure	18.5 (13.9, 23.1)	37 (28, 45)		11.8 (8.0, 15.6)	37 (25, 49)
Smoking: differential vulnerability	4.6 (-1.9, 11.0)	9 (-4, 22)		5.3 (0.0, 10.6)	9 (-4, 22)
**Body Mass Index**					
Total effect of low education	33.2 (28.9, 37.4)	100		14.5 (11.2, 17.8)	100
Direct effect of low education	32.4 (28.1, 36.7)	98 (85, 112)		15.8 (12.4, 19.2)	109 (84, 141)
Indirect effect of low education	0.8 (-3.7, 5.3)	2 (-11, 16)		-1.3 (-5.0, 2.5)	-9 (-36, 17)
BMI: differential exposure	3.5 (-0.3, 7.2)	10 (-1, 21)		0.5 (-2.3, 3.3)	4 (-17, 21)
BMI: differential vulnerability	-2.7 (-8.5, 3.1)	-8 (-26, 9)		-1.8 (-6.5, 2.9)	-13 (-49, 19)
**Physical activity**					
Total effect of low education	46.7 (42.5, 50.9)	100		25.5 (22.2, 28.8)	100
Direct effect of low education	28.4 (24.0, 32.8)	61 (52, 70)		13.7 (10.3, 17.1)	54 (41, 66)
Indirect effect of low education	18.3 (13.7, 22.9)	39 (29, 50)		11.8 (8.2, 15.5)	46 (32, 63)
Physical activity: differential exposure	13.4 (9.2, 17.6)	29 (20, 37)		6.9 (3.8, 9.9)	27 (16, 38)
Physical activity: differential vulnerability	4.9 (-1.3, 11.1)	10 (-3, 23)		5.0 (0.2, 9.7)	20 (1, 38)

The models adjusted for age (as timescale), race/ethnicity, marital status, survey year, and the lifestyle factors; for simplicity, only the effect of low education (relative to high education) is presented.CI: confidence interval.
[a] Proportion mediated is the ratio between the effect and the total effect x 100

© 2022 Puka K et al. *JAMA Health Forum.*

eTable 10. Participant Characteristics at Baseline, Stratified by Education

	Complete sample		Low education	Medium education	High education
Sample size, n	415,764		188,845	115,641	111,278
Sex, n female	229,994 (55%)		105,314 (56%)	66,399 (57%)	58,281 (52%)
Age at baseline, mean yrs (SD)	49.4 (15.8)		51.9 (16.4)	47.7 (15.0)	47.0 (14.7)
Follow-up, mean yrs (SD)	8.8 (5.2)		8.9 (5.2)	8.8 (5.3)	8.7 (5.3)
Person-years	3,672,747		1,682,680	1,019,844	970,223
All-cause deaths, n (%)	49,096 (12%)		31,544 (17%)	10,777 (9%)	6,775 (6%)
Death rate, per 10,000 person-years	133.7		187.5	105.7	69.8
Alcohol use, %					
Never drinker	31		40	26	21
Former drinker	7		9	7	5
Low risk	58		47	63	72
Moderate risk	2		2	2	2
High risk	1		2	1	1
Smoking, %					
Never smoker	55		50	52	67
Former smoker	24		24	25	23
Current some day smoker	4		5	5	3
Current everyday smoker	17		22	18	7
BMI, %					
Underweight	2		2	1	2
Healthy weight	35		31	33	43
Overweight	36		37	36	36
Obese	27		31	30	20
Physical activity, %					
Active	42		30	46	59
Somewhat active	18		16	19	19
Sedentary	40		53	35	22
Ethnicity, %					
Black, non-Hispanic	14		16	16	10
Hispanic	17		24	13	7
White, non-Hispanic	64		56	67	74
Other, non-Hispanic	5		3	4	9
Married/cohabitating, %	56		54	54	61

yrs: years ; SD: standard deviation.

© 2022 Puka K et al. *JAMA Health Forum.*

eTable 11. Results of Additive Hazard Models; Among All Participants

	**All Participants**
	Additional deaths per 10,000 person-years (95% CI)
**Alcohol Use x Education**	
High education, Category I drinking	reference
Low education, Category I drinking	43.0 (40.1, 45.9)
High education, Category III drinking	69.8 (43.1, 96.5)
Low education, Category III drinking	142.3 (126.5, 158.1)
Additional deaths due interaction	29.5 (-1.6, 60.6)
**Smoking x Education**	
High education, never smoker	reference
Low education, never smoker	20.1 (17.1, 23.2)
High education, everyday smoker	65.7 (58.0, 73.5)
Low education, everyday smoker	124.6 (120.3, 128.9)
Additional deaths due interaction	**38.7 (29.6, 47.8)**
**Body Mass Index x Education**	
High education, healthy weight	reference
Low education, healthy weight	64.1 (59.9, 68.3)
High education, obese	6.3 (1.6, 10.9)
Low education, obese	66 (61.7, 70.2)
Additional deaths due interaction	-4.4 (-11.3, 2.4)
**Physical Activity x Education**	
High education, active	reference
Low education, active	32.9 (29.5, 36.2)
High education, sedentary	44.9 (40.0, 49.8)
Low education, sedentary	109.1 (105.6, 112.6)
Additional deaths due interaction	**31.3 (24.9, 37.7)**

The model was adjusted for age (as timescale), sex, race/ethnicity, marital status, and survey year. Bolded text highlights significant interactions. CI: confidence interval.

© 2022 Puka K et al. *JAMA Health Forum.*

eTable 12. Results of Causal Mediation Analyses; Among All Participants

	All Adults	
	Additional deaths per 10,000 person-years (95% CI)	Proportion mediated % (95% CI) [a]
Total effect of low education	66.4 (64.8, 68.1)	100
Direct effect of low education	19.7 (18.0, 21.4)	30 (27, 32)
Indirect effect of low education	46.7 (44.9, 48.6)	70 (67, 74)
Alcohol use: differential exposure	6.6 (5.4, 7.7)	10 (8, 12)
Alcohol use: differential vulnerability	2.9 (1.4, 4.4)	4 (2, 7)
Smoking: differential exposure	14.4 (13.2, 15.6)	22 (20, 23)
Smoking: differential vulnerability	3.1 (1.6, 4.6)	5 (3, 7)
BMI: differential exposure	1.2 (0.1, 2.4)	2 (0, 4)
BMI: differential vulnerability	-0.5 (-2.0, 0.9)	-1 (-3, 1)
Physical activity: differential exposure	14.7 (13.5, 15.9)	22 (20, 24)
Physical activity: differential vulnerability	4.4 (2.9, 5.9)	7 (4, 9)

The model adjusted for age (as timescale), race/ethnicity, marital status, and survey year; for simplicity, only the effect of low education (relative to high education) is presented. CI: confidence interval.

[a] Proportion mediated is the ratio between the effect and the total effect x 100

© 2022 Puka K et al. *JAMA Health Forum.*
